# Metabolic Actions of a Supplement of Ilex Paraguariensis (An Extract of the Leaf Standardized to 2% I-Deoxinojirimcina), White Mulberry and Chromium Picolinate in Nondiabetic Subects with Dysglycemia: A Randomized Trial

**DOI:** 10.3390/life11070709

**Published:** 2021-07-18

**Authors:** Giuseppe Derosa, Angela D’Angelo, Pamela Maffioli

**Affiliations:** 1 Department of Internal Medicine and Therapeutics, University of Pavia, 27100 Pavia, Italy; labmedmol@smatteo.pv.it; 2 Centre of Diabetes, Metabolic Diseases, and Dyslipidemias, University of Pavia, 27100 Pavia, Italy; pamelamaffioli@hotmail.it; 3 Regional Centre for Prevention, Surveillance, Diagnosis and Treatment of Dyslipidemias and Atherosclerosis, Fondazione IRCCS Policlinico San Matteo, 27100 Pavia, Italy; 4 Laboratory of Molecular Medicine, University of Pavia, 27100 Pavia, Italy

**Keywords:** Ilex paraguariensis, white mulberry, chromium picolinate, impaired glycemia

## Abstract

Aim: To prove if a nutraceutical containing Ilex paraguariensis (*Ilex L.* spp. Aquifoliales) (an extract of the leaf standardized to 2% I-deoxinojirimcina), white mulberry (*Morus* spp., Moraceae), and chromium picolinate can be effective in improving glycemic status in subject with dysglycemia. Methods: We randomized patients to consume placebo or the nutraceutical, self-administered once a day, one tablet at breakfast, for 3 months. Results: A reduction in fasting plasma glucose, postprandial glucose, and glycated hemoglobin was observed with the nutraceutical combination, both compared to baseline and placebo. Data suggested a decrease in the Homeostasis Model Assessment index with the nutraceutical, both compared to baseline and placebo. The M value, an index of insulin sensitivity, obtained after nutraceutical treatment was higher compared to baseline. We recorded a decrease in total cholesterol, low-density lipoprotein-cholesterol, and triglycerides with the nutraceutical combination compared to baseline and placebo. A decrease in high-sensitivity C-reactive protein was observed with the nutraceutical combination compared to baseline and placebo. Conclusions: A nutraceutical containing Ilex paraguariensis, white mulberry, and chromium picolinate can be helpful in improving glycemic status and lipid profile in dysglycemic subjects.

## 1. Introduction

The condition of euglycemia is characterized by levels of fasting plasma glucose (FPG) >70 mg/dL and <100 mg/dL. Euglycemia is usually maintained by the balance of two antagonist hormones: glucagon, with a glucose-elevating action, secreted by pancreatic α-cells; and insulin, with a glucose-lowering effect, produced, instead, by pancreatic β-cells. Muscular cells, adipose tissue, and liver cells have receptors for insulin and glucagon. Hyperglycemia has been related to several complications, including macrovascular and microvascular complications [1]. Moreover, postprandial glucose (PPG) is an independent cardiovascular risk factor and a powerful inducer of endothelial damage [2,3]. Dysglycemia is defined by FPG ≥100 mg/dL but <126 mg/dL and includes different conditions, such as impaired fasting glucose (IFG), impaired glucose tolerance (IGT), and type 2 diabetes mellitus. According to the different glycemic status, the therapeutic intervention involves a change in lifestyle, in addition to nutraceutical agents in the case of IFG or IGT, and a combined behavioral intervention and pharmacological drug treatment in the case of type 2 diabetes mellitus. Among nutraceuticals [4], it has been observed that consumption of Ilex paraguariensis (*Ilex L.* spp. Aquifoliales) improves the glycemic and lipid profile in patients with type 2 diabetes mellitus treated with oral hypoglycemic agents (metformin and sulfonylureas) [5]. Previous studies have demonstrated that the consumption of Ilex paraguariensis alone improved lipid profile in normolipidemic, dyslipidemic, and hypercholesterolemic subjects [6] and also induced a decrease in body weight in overweight subjects [7]. 

Mulberry (*Morus* spp., Moraceae) is a multifunctional plant. Mulberry contains a latex sap that is toxic to humans; however, the leaves and fruits contain numerous chemical constituents that should be helpful for treating several diseases. I-deoxinojirimcina (DNJ), phenolics, and flavonoids are the main functional compounds. The fresh fruits are edible and harvested for food production, including juice, jam, and jelly. Furthermore, the leaves are highly palatable. Different parts of the mulberry tree, such as root bark, leaves, and fruits, seem to have a beneficial effect in the treatment of fever, cough, hyperlipidemia, hypertension, and hyperglycemia [8]. Several studies conducted in humans suggested that consumption of white mulberry *(Morus alba*) leaves reduces fasting plasma glucose (FPG) and glycated hemoglobin (HbA_1c_) values in patients with type 2 diabetes mellitus not well controlled by conventional sulfonylurea and/or alfa-inhibitory glucosidase therapy [9].

Chromium is an important cofactor for many actions of insulin metabolism. Chromium plays a role in insulin binding to its receptors in striated muscle cells, adipocytes, and hepatocytes and promotes the phosphorylation of receptors, improving glucose tolerance [10]. A review reported that chromium picolinate intake is effective in preventing or delaying the onset of type 2 diabetes, as it reduces hyperglycemia, total cholesterol (TC) and triglyceride (Tg) levels, body weight, and fat mass [11]. In the literature, data are presented on the use of these substances combined, suggesting a positive effect on glycemia [12].

On these bases, the primary purpose of this study was to evaluate if Glicoset^®^ 1000, a nutraceutical containing Ilex paraguariensis (an extract of the leaf standardized to 2% I- deoxinojirimcina), white mulberry, and chromium picolinate, can be useful in improving glycemic status in dysglycemic patients. The secondary purpose included the changes in lipid profile and inflammatory status.

## 2. Materials and Methods

### 2.1. Study Design

We conducted a 3-month, randomized, double-blind, placebo-controlled clinical study. The trial was conducted at the Centre of Diabetes and Metabolic Diseases, Department of Internal Medicine and Therapeutics, University of Pavia and Fondazione IRCCS Policlinico San Matteo, PAVIA, Italy.

The study protocol was approved by the institutional review board (Fondazione IRCCS Policlinico San Matteo, P.le Golgi, 19-27100-Pavia-Italy) and was conducted in accordance with the 1994 Declaration of Helsinki [13], its amendments, and the Code of Good Clinical Practice. TRIAL REGISTRATION: ClinicalTrials.gov NCT04107922.

We required patients’ written informed consent to participate in this study. Consent was obtained after a full explanation of the scheduled procedures.

### 2.2. Patients

We included in the trial patients ≥18 years old, males and females, affected by IFG or IGT, and not taking a glucose-lowering medication (both pharmaceuticals or nutraceutical agents). Investigators identified suitable patients from a review of case notes and/or computerized clinic registers and contacted them in person or by telephone. 

The trial excluded patients with type 1 or type 2 diabetes mellitus, impaired hepatic or renal function, or gastrointestinal disorders. Patients with current or previous evidence of ischemic heart disease, heart failure, or stroke were also excluded. Patients with a weight change of >3 kg during the preceding 3 months or malignancy and significant neurological or psychiatric disturbances, including alcohol or drug abuse, were not included in the study. 

### 2.3. Treatments

We randomized patients to take placebo or Glicoset^®^ 1000 for 3 months. Glicoset^®^ 1000 and placebo were self-administered once a day, one tablet at breakfast. 

To ensure the double-blind status of the study, both Glicoset^®^ 1000 and placebo were supplied as identical, opaque tablets in coded bottles (Table 1). Investigators conducted randomization using a drawing of envelopes containing randomization codes. The codes were prepared by a statistician. Treatment compliance was assessed at the randomization and at the end of the study by counting the number of pills returned. All unused tablets were retrieved for inventory. All treatments were provided free of charge, and no compensation was given to the patients for participation in this study.

### 2.4. Assessments

Before starting the study, all patients underwent an initial screening assessment that included medical history, a physical examination, vital signs (blood pressure and heart rate), a 12-lead electrocardiogram, measurements of height and body weight, and calculation of body mass index (BMI). We also assessed FPG, PPG, HbA_1c_, fasting plasma insulin (FPI), Homeostasis Model Assessment index (HOMA index), TC, low-density lipoprotein-cholesterol (LDL-C), high-density lipoprotein-cholesterol (HDL-C), Tg, aspartate aminotransferase (AST), alanine aminotransferase (ALT), and high-sensitivity C-reactive protein (Hs-CRP).

We evaluated each parameter at baseline and at the end of the study. Moreover, at baseline and at the study end, patients underwent an oral glucose tolerance test (OGTT) and a euglycemic hyperinsulinemic clamp. All plasmatic variables were determined after a 12 h overnight fast, with the exception of PPG. Venous blood samples were drawn by a research nurse for all patients between 8:00 a.m. and 9:00 a.m. We used plasma obtained by the addition of 1 mg/mL Na_2_-EDTA, centrifuged at 3000× *g* for 15 min at 4 °C. Immediately after centrifugation, the plasma samples were frozen and stored at −80 °C for ≤3 months. Laboratory technicians drew blood samples, and the biologist responsible for the laboratory performed the assays. All measurements were performed in a central laboratory. Body mass index was calculated by the investigators as weight in kilograms divided by the square of height in meters. Plasma glucose was assayed using the glucose-oxidase method (GOD/PAP, Roche Diagnostics, Mannheim, Germany) with intra- and interassay coefficients of variation (CsV) <2% [14]. Plasma insulin was assayed with the Phadiaseph insulin radioimmunoassay (RIA) (Pharmacia, Uppsala, Sweden) by using a second antibody to separate the free and antibody-bound 125 I-insulin (intra- and interassay CsV of 4.6% and 7.3%, respectively) [15]. The HOMA-IR index was calculated as the product of basal glucose (mmol/L) and insulin levels (μU/mL) divided by 22.5 [16,17]. Total cholesterol and Tg levels were determined using fully enzymatic techniques [18,19] on a clinical chemistry analyzer (Hitachi 737; Hitachi, Tokyo, Japan); intra- and interassay CsV were 1.0% and 2.1% for TC measurement and 0.9% and 2.4% for Tg measurement, respectively. HDL-C level was measured after the precipitation of plasma apo B-containing lipoproteins with phosphotungstic acid [20]; intra- and interassay CsV were 1.0% and 1.9%, respectively. LDL-C level was calculated using the Friedewald formula [21]. Transaminases were evaluated in the central lab according to the International Federation of Clinical Chemistry (IFCC) method [22,23]. High-sensitivity C-reactive protein was measured with the use of latex-enhanced immunonephelometric assays on a BN II analyzer (Dade Behring, Newark, DE, USA). The intra- and interassay CsV were 5.7% and 1.3%, respectively [24].

### 2.5. Safety Measurements

Adverse events were evaluated at baseline and at the study end with an accurate interview conducted by the investigators. Investigators also compared clinical and laboratory values at the study end with baseline levels. 

### 2.6. Oral Glucose Tolerance Test and Glucose Clamp Technique

All subjects drank a glass of water (200 mL) in which 75 g of glucose had been dissolved over a period of 5 min in the morning between 8 and 9 a.m. after a 12 h fast and after dietary assessment to ensure a carbohydrate intake >150 g/day over the previous 3 days [25]. Normal physical activity was allowed over the previous 3 days. No smoking was allowed during the test. Blood samples were collected in EDTA-containing tubes (Becton Dickinson, CEDEX, Meylan, France) through a venous catheter from an antecubital vein immediately before and at 120 min after the glucose load for the measurement of the considered parameters of the study. On the basis of the results recorded 2 h after the OGTT, we diagnosed patients as being affected by IFG, IGT, or type 2 diabetes mellitus. In particular:

- IFG was defined by glycemia at 120 min from OGTT < 140 mg/dL;
- IGT was defined by glycemia at 120 min from OGTT between 140 mg/dL and 199 mg/dL;
- Type 2 diabetes mellitus was defined by glycemia at 120 min from OGTT ≥ 200 mg/dL.

A euglycemic hyperinsulinemic clamp was performed to assess insulin sensitivity [26]. 

Clamps were performed before randomization and at the end of the study. At 9:00 a.m., after the patients had fasted for 12 h overnight, an indwelling cannula (18-gauge polyethylene cannula; Venflon, Viggo, Helsingborg, Sweden) was placed into an antecubital vein for infusion of glucose and insulin. To obtain arterialized venous blood samples, an indwelling catheter was inserted in a retrograde fashion into a dorsal hand or wrist vein and maintained in a heated box at 70 °C. In the contralateral arm, a second cannula was introduced anterogradely in an antecubital vein of the forearm for the variable infusion of 20% glucose (L.I.M., Biondustria, Novi Ligure, AL, Italy) and insulin (1 mU min^−1^ Kg^−1^, Humulin R, Eli Lilly, Indianapolis, IN, USA) using a Terumo microinfusion pump (TE-371 TIVA, Terumo Corporation, Tokyo, Japan). Arterialized blood samples were collected every 5 min to determine glucose concentration (EML 105, Radiometer, Copenhagen, Denmark). The amount of glucose infused was adjusted to maintain euglycemia at 90 mg/dL. During the euglycemic hyperinsulinemic clamp, the M value was calculated based on the last 30 min (steady state) and after adjustments of the steady-state insulin concentration (M/I). 

### 2.7. Statistical Analysis

Considering as clinically significant a difference of at least 10% compared to the baseline and an alpha error of 0.05, the actual sample size was adequate to obtain a power higher than 0.80 for all measured variables. Patients were included in the tolerability analysis if they had received ≥1 dose of trial treatment after randomization and had undergone a subsequent tolerability observation. Continuous variables were tested using a two-way repeated-measures analysis of variance (ANOVA). Intervention effects were adjusted for additional potential confounders using analysis of covariance. Analysis of variance was also used to assess the significance within and between groups. The null hypothesis that the expected mean glycemia change from the end of the study did not differ significantly between placebo and the nutraceutical compound was tested using a two-way repeated-measures analysis of variance (ANOVA) model [27]. A 1-sample *t* test was used to compare values obtained before and after treatment administration; 2-sample *t* tests were used for between-group comparisons. Statistical analysis of data was performed using the Statistical Package for Social Sciences software version 24.0 (SPSS Inc., Chicago, IL, USA). Data are presented as the mean and standard deviation (SD). A *p* < 0.05 was considered statistically significant.

## 3. Results

### 3.1. Study Sample

**Figure 1:**
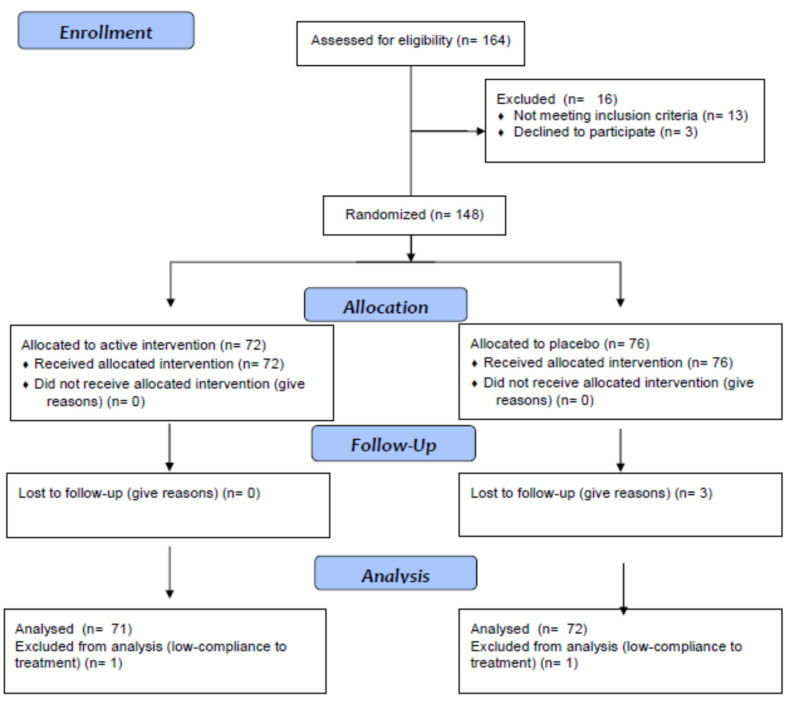
CONSORT 2010 flow diagram.

One hundred and forty-eight patients were enrolled in the trial; 72 were randomized to Glicoset^®^ 1000 and 76 to placebo. One hundred and forty-three subjects completed the study; five patients did not complete the study because they were not compliant to treatment (at least 80% of compliance to treatment was required) or were lost to follow-up (Figure 1). The first patient was enrolled on 1 October 2018, while the last patient was enrolled on 31 May 2019. Follow-up of the last patient enrolled was completed on 31 August 2019. Patient population characteristics at the study start and at the end of the study are shown in Table 2 and Table 3.

### 3.2. Anthropometric Parameters and Glycemic Metabolism

We did not record any significant variations regarding BMI or circumferences with either treatment (Table 3). There was a decrease of FPG, PPG, and HbA_1c_ with the nutraceutical combination, both compared to baseline and placebo (*p* < 0.05, for both). The HOMA index decreased with the nutraceutical combination compared to baseline (*p* < 0.05) and placebo (*p* < 0.05) (Table 3).

### 3.3. OGTT Results

**Figure 2:**
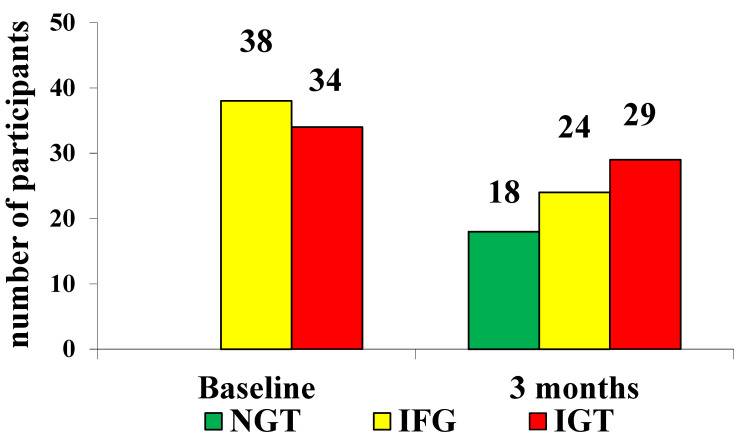
Results after the OGTT at baseline and after 3 months in patients treated with Glicoset^®^ 1000 (NGT: normal glucose tolerance; IFG: impaired fasting glucose; IGT: impaired glucose tolerance).

**Figure 3:**
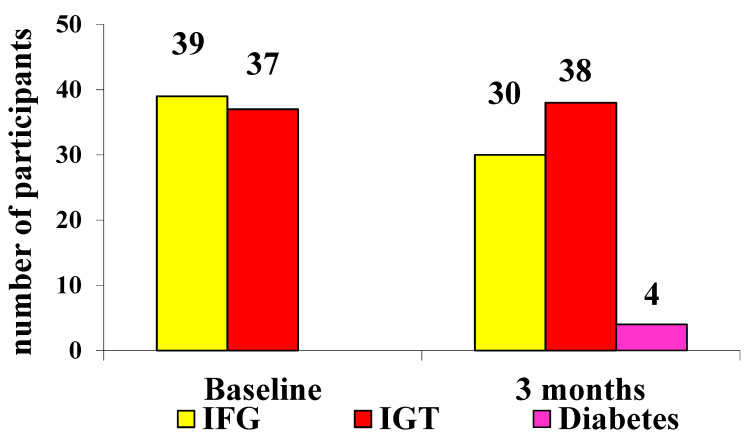
Results after OGTT at baseline and after 3 months in patients treated with placebo (IFG: impaired fasting glucose; IGT: impaired glucose tolerance).

At baseline, we had 52.8% of patients with IFG in the nutraceutical group vs. 51.3% in the placebo group (*p* not significant) and 47.2% of patients with IGT in the nutraceutical group and 48.7% in the placebo group (*p* not significant). After 3 months, 25.3% of subjects returned to a normal glycemic status in the nutraceutical group vs. 0 patients in the placebo group (*p* < 0.05). At the study end, 33.8% of patients were classified as IFG in the nutraceutical group vs. 41.7% in the placebo group (*p* < 0.05). In the nutraceutical group, 40.8% were classified as IGT vs. 52.8% in the placebo group (*p* < 0.01). In the placebo group, 5.6% developed type 2 diabetes mellitus vs. 0 patients in the nutraceutical group (Table 2, Table 4 and Table 5; Figure 2 and Figure 3). 

### 3.4. M Value during the Clamp Technique

Nutraceutical treatment, but not placebo, achieved a higher M value at the end of the study compared to baseline (*p* < 0.05 vs. baseline). 

At the study end, a higher number of patients returned to normal insulin sensitivity (78%) with the nutraceutical compared to placebo. Moreover, 22% of patients reached an M value ≥ 4 and <7.5 mg/kg/min, and no patients had insulin resistance at the end of the study in the nutraceutical treatment (Table 6).

### 3.5. Lipid Profile

We recorded a reduction of TC, LDL-C, and Tg with the nutraceutical combination, both compared to baseline and placebo (*p* < 0.05 for both) (Table 3).

### 3.6. Cytokines

The nutraceutical combination gave a decrease of Hs-CRP compared to baseline (*p* < 0.05) and placebo (*p* < 0.05 for both) (Table 3).

### 3.7. Safety and Treatment Acceptance

No significant adverse events were reported. No differences were recorded among groups regarding acceptance of treatment that was well tolerated.

## 4. Discussion

In the current study, we recorded that a nutraceutical containing Ilex paraguariensis (an extract of the leaf standardized to 2% I-deoxinojirimcina), white mulberry, and chromium picolinate improved glycemic status in dysglycemic patients and, in particular, reduced FPG, PPG, and HbA_1c_. The mechanism by which Ilex paraguariensis could improve dysglycemia was suggested by Arcari et al., which showed a modulatory effect on different genes (Akt2, Irs1, Irs2, Pi3kca, Pi3kcg, and Pdk1) involved in insulin resistance [28]. The hypoglycemic action of the white mulberry leaves, instead, is due to the presence of iminosugars such as I-deoxinojirimcina (DNJ), a powerful intestinal alfa-glucosidase inhibitor, which reduces the rise of PPG and PPI, with an acarbose-like mechanism [29]. We already conducted a study about the effects of a hypoglycemic nutraceutical containing Ascophyllum nodosum and Fucus vesiculosus in a ratio of 95/5 and chromium picolinate [30]. Ascophyllum nodosum and Fucus vesiculosus act in an acarbose-like mechanism, and also in this case, we recorded a similar reduction in HbA_1c_, FPG, PPG, and the HOMA index compared to placebo, suggesting that reducing glucose absorption can be a valid option to prevent diabetes [30]. 

Regarding the effect of the nutraceutical on lipid profile, our data are in line with de Morais et al. [6]. The authors enrolled 102 individuals, 15 normolipidemic, 57 dyslipidemic, and 30 hypercholesterolemic subjects, on long-term statin therapy. The investigators administered 330 mL, 3 times/day, of green or roasted Ilex paraguariensis leaf infusions for 40 days. In normolipidemic subjects, Ilex paraguariensis consumption decreased LDL-C by 8.7% and in dyslipidemic individuals by 8.6%. The consumption of Ilex paraguariensis by hypercholesterolemic individuals on statin therapy achieved an additional 13.1% reduction in LDL-C after 40 days and increased HDL-C by 6.2%. 

Matsumoto et al. investigated the effects of Ilex paraguariensis supplementation on plasma susceptibility to oxidation and on antioxidant enzyme gene expression in healthy women [31]. The authors showed that regular consumption of Ilex paraguariensis may empower body antioxidant defense. This is in line with our study, as we did not specifically evaluate antioxidant enzyme gene expression, but we reported a decrease in Hs-CRP, which could suggest an improvement in inflammatory status. 

Of course, our study has several limitations, such as the short duration of the trial; moreover, we considered just a few inflammatory markers and, in particular, Hs-CRP. Furthermore, we did not observe if the nutraceutical agent effects were maintained after the trial ending and the interruption of treatment. The effects of the amount of exercise and diet of the subjects were not evaluated given the short study follow-up. Subject compliance regarding the pills was obtained by counting the number of pills left just at the end of the study and not during the trial. Finally, treatment tolerability was assessed just at the end of the study.

## 5. Conclusions

We can conclude that a nutraceutical containing Ilex paraguariensis, white mulberry, and chromium picolinate could improve glycemic status and lipid profile in subjects with IFG or IGT. This nutraceutical could be a valid option, in addition to diet and physical activity, to prevent type 2 diabetes in patients with dysglycemia.

## Figures and Tables

**Table 1:** Composition of Glicoset^®^ 1000 and placebo.

**Ingredients of Glicoset^®^ 1000**	**Daily intake (1 Tablet)**
Chromium picolinate	100 mcg (250% RDD)
Ilex paraguariensis	1000 mg
Morus alba (2% I-deoxinojirimcina)	50 mg of which 1 mg DNJ
Silicon dioxide	13 mg
Magnesium stearate	13 mg
Dicalcium phosphate	139 mg
Microcrystalline cellulose	84.2 mg
**Ingredients of Placebo**	**Daily intake (1 Tablet)**
Silicon dioxide	13 mg
Magnesium stearate	13 mg
Dicalcium phosphate	600 mg
Microcrystalline cellulose	609 mg
E172 (iron oxides, a food coloring)	65 mg

RDD: recommended daily dose; DNJ: I-deoxinojirimcina.

**Table 2:** Baseline and 3-month data of patients during Glicoset^®^ 1000 treatment and placebo.

Parameters	Glicoset^®^ 1000		Placebo	
	Baseline	3 Months	Baseline	3 Months
Patients	72	71	76	72
M/F	35/37	35/36	38/38	35/37
Smoking status (M/F)	14/16	14/16	13/12	12/11
IFG (M/F (%))	20/18 (52.8)	13/11 (33.8)	21/18 (51.3)	16/14 (41.7)
IGT (M/F (%))	15/19 (47.2)	13/16 (40.8)	17/20 (48.7)	19/19 (52.8)
EU from IFG (M/F (%))	-	7/6 (18.3)	-	0/0
EU from IGT (M/F (%))	-	2/3 (7.0)	-	0/0
IGT from IFG (M/F (%))	-	0/0	-	3/3 (8.3)
D from IFG (M/F (%))	-	0/0	-	0/0
D from IGT (M/F (%))	-	0/0	-	1/3 (5.6)
Lost to FU from IFG (M/F (%))	-	0/1 (1.4)	-	2/1 (4.2)
Lost to FU from IGT (M/F (%))	-	0/0	-	0/1 (1.4)

M: males; F: females; IFG: impaired fasting glucose; IGT: impaired glucose tolerance; EU: euglycemia; D: diabetes; FU: follow-up.

**Table 3:** Baseline and 3-month data of patients during Glicoset^®^ 1000 treatment and placebo.

Parameters	Glicoset^®^ 1000		Placebo	
	Baseline	3 Months	Baseline	3 Months
Patients	72	71	76	72
M/F	35/37	35/36	38/38	35/37
Age (years)	53.8 ± 6.4	-	52.7 ± 6.1	-
Smoking status (M/F)	15/16	15/15	13/16	12/14
Height (cm)	1.68 ± 0.04	1.68 ± 0.04	1.69 ± 0.05	1.69 ± 0.05
Weight (Kg)	79.2 ± 9.8	79.0 ± 9.6	78.4 ± 9.4	78.6 ± 9.5
BMI (Kg/m^2^)	28.1 ± 2.4	28.1 ± 2.4	27.4 ± 2.1	27.5 ± 2.2
WC (cm)	90.3 ± 3.3	90.3 ± 3.3	89.7 ± 3.1	89.8 ± 3.2
HC (cm)	87.5 ± 2.8	87.4 ± 2.7	87.1 ± 2.6	87.3 ± 2.8
AC (cm)	99.2 ± 3.4	99.2 ± 3.3	98.3 ± 3.1	98.4 ± 3.2
FPG (mg/dL)	114.2 ± 8.6	102.2 ± 5.9 *^	113.1 ± 8.3	115.7 ± 12.8
PPG (mg/dL)	132.9 ± 9.8	120.4 ± 5.0 *^	129.8 ± 5.2	132.1 ± 5.6
HbA_1c_ (%)	5.9 ± 0.3	5.5 ± 0.1 *^	5.7 ± 0.2	5.8 ± 0.3
FPI (μU/mL)	9.1 ± 5.3	9.0 ± 5.2	8.9 ± 5.1	9.0 ± 5.2
HOMA index	2.53 ± 0.9	2.38 ± 0.6 *^	2.48 ± 0.9	2.58 ± 0.9
TC (mg/dL)	218.2 ± 17.4	197.4 ± 15.3 *^	214.7 ± 16.3	217.1 ± 16.8
LDL-C (mg/dL)	148.8 ± 20.2	130.7 ± 17.5 *^	146.7 ± 19.7	147.5 ± 19.9
HDL-C (mg/dL)	43.2 ± 4.5	43.0 ± 4.2	42.5 ± 4.2	42.7 ± 4.4
Tg (mg/dL)	131.1 ± 39.1	118.5 ± 31.8 *^	127.3 ± 38.4	134.6 ± 38.7
AST (UI/L)	18.1 ± 8.6	17.5 ± 8.3	19.2 ± 9.2	18.7 ± 8.9
ALT (UI/L)	22.0 ± 12.1	21.6 ± 11.7	22.8 ± 12.6	24.1 ± 12.9
Hs-CRP (mg/L)	1.1 ± 0.6	1.0 ± 0.5	1.0 ± 0.5	1.2 ± 0.7

Data are expressed as the mean ± standard deviation. * *p* < 0.05 vs. baseline; ^ *p* < 0.05 vs. placebo. M: males; F: females; BMI: body mass index; WC: waist circumference; HC: hip circumference; AC: abdominal circumference; FPG: fasting plasma glucose; PPG: postprandial glucose; HbA_1c_: glycated hemoglobin; FPI: fasting plasma insulin; HOMA index: Homeostasis Model Assessment index; TC: total cholesterol; LDL-C: low-density lipoprotein-cholesterol; HDL-C: high-density lipoprotein-cholesterol; Tg: triglyceride; AST: aspartate aminotransferase; ALT: alanine aminotransferase; Hs-CRP: high-sensitivity C-reactive protein.

**Table 4:** OGTT results at baseline.

		Glicoset^®^ 1000		Placebo	
	Time (Minutes)	Glycemia (mg/dL)	Subjects n (M/F)	Glycemia (mg/dL)	Subjects n (M/F)
IFG	0	106.7 ± 5.8	38 (20/18)	106.5 ± 5.4	39 (21/18)
	120	122.4 ± 10.5		123.4 ± 11.2	
IGT	0	108.6 ± 6.9	34 (15/19)	109.7 ± 7.5	37 (17/20)
	120	162.1 ± 15.7		158.9 ± 14.3	

M: males; F: females; IFG: impaired fasting glucose; IGT: impaired glucose tolerance.

**Table 5:** OGTT results at the end of the study.

		Glicoset^®^ 1000		Placebo	
	Time (minutes)	Glycemia (mg/dL)	Subjects n (M/F)	Glycemia (mg/dL)	Subjects n (M/F)
IFG	0	103.6 ± 3.0	24 (13/11)	105.4 ± 4.8	30 (16/14)
	120	114.2 ± 6.5		118.5 ± 11.6	
IGT	0	105.3 ± 5.1	29 (13/16)	108.2 ± 6.9	38 (19/19)
	120	149.4 ± 8.3		153.7 ± 11.3	

Data are the mean ± SD. n: number of subjects; IFG: impaired fasting glucose, IGT: impaired glucose tolerance.

**Table 6:** Baseline and 3-month clamp data (M value) in patients treated with Glicoset^®^ 1000 or placebo.

	N Baseline	N End of Treatment	Baseline	End of Treatment	Delta End of Treatment vs. Baseline
Glicoset^®^ 1000	72	71	6.19 ± 0.88	8.28 ± 1.27 *°	2.09 ± 0.92
Placebo	76	71	6.04 ± 0.52	5.98 ± 0.83	0.06 ± 0.05

Data are expressed as the mean ± SD (standard deviation). * *p* < 0.05 vs. baseline; ° *p* < 0.05 vs. placebo. Definition of insulin sensitivity: Normal insulin sensitivity: M value ≥ 7.5 mg/kg/min. Impaired glucose tolerance: M value ≥ 4 and < 7.5 mg/kg/min. Insulin resistance: M value < 4 mg/kg/min.

## Data Availability

The processed data supporting reported results cannot be shared at this time due to ethical reasons.

## Abbreviations

M: males; F: females; IFG: impaired fasting glucose; IGT: impaired glucose tolerance; EU: euglycemia; D: diabetes; FU: follow-up; BMI: body mass index; WC: waist circumference; HC: hip circumference; AC: abdominal circumference; FPG: fasting plasma glucose; PPG: postprandial glucose; HbA1c: glycated hemoglobin; FPI: fasting plasma insulin; HOMA index: Homeostasis Model Assessment index; TC: total cholesterol; LDL-C: low-density lipoprotein-cholesterol; HDL-C: high-density lipoprotein-cholesterol; Tg: triglyceride; AST: aspartate aminotransferase; ALT: alanine aminotransferase; Hs-CRP: high-sensitivity C-reactive protein.
